# Proteomics Analysis of the Protective Effect of Polydeoxyribonucleotide Extracted from Sea Cucumber (*Apostichopus japonicus*) Sperm in a Hydrogen Peroxide-Induced RAW264.7 Cell Injury Model

**DOI:** 10.3390/md22070325

**Published:** 2024-07-21

**Authors:** Zhiqiang Shu, Yizhi Ji, Fang Liu, Yuexin Jing, Chunna Jiao, Yue Li, Yunping Zhao, Gongming Wang, Jian Zhang

**Affiliations:** 1 Department of Food Science and Technology, Shanghai Ocean University, Shanghai 200120, China; s13970318519@163.com (Z.S.);; 2 Shandong Marine Resource and Environment Research Institute, Yantai 264006, China; 3 Yantai Key Laboratory of Quality and Safety Control and Deep Processing of Marine Food, Yantai 264006, China

**Keywords:** sea cucumber sperm, polydeoxyribonucleotide, antioxidant, proteomics, RAW264.7 cell, oxidative stress

## Abstract

Sea cucumber viscera contain various naturally occurring active substances, but they are often underutilized during sea cucumber processing. Polydeoxyribonucleotide (PDRN) is an adenosine A_2A_ receptor agonist that activates the A_2A_ receptor to produce various biological effects. Currently, most studies on the activity of PDRN have focused on its anti-inflammatory, anti-apoptotic, and tissue repair properties, yet relatively few studies have investigated its antioxidant activity. In this study, we reported for the first time that PDRN was extracted from the sperm of *Apostichopus japonicus* (AJS-PDRN), and we evaluated its antioxidant activity using 2,2-diphenyl-1-picrylhydrazyl (DPPH), 2,2′-azino-bis-3-ethylbenzothiazoline-6-sulphonic acid (ABTS), and hydroxyl radical scavenging assays. An in vitro injury model was established using H_2_O_2_-induced oxidative damage in RAW264.7 cells, and we investigated the protective effect of AJS-PDRN on these cells. Additionally, we explored the potential mechanism by which AJS-PDRN protects RAW264.7 cells from damage using iTRAQ proteomics analysis. The results showed that AJS-PDRN possessed excellent antioxidant activity and could significantly scavenge DPPH, ABTS, and hydroxyl radicals. In vitro antioxidant assays demonstrated that AJS-PDRN was cytoprotective and significantly enhanced the antioxidant capacity of RAW264.7 cells. The results of GO enrichment and KEGG pathway analysis indicate that the protective effects of AJS-PDRN pretreatment on RAW264.7 cells are primarily achieved through the regulation of immune and inflammatory responses, modulation of the extracellular matrix and signal transduction pathways, promotion of membrane repair, and enhancement of cellular antioxidant capacity. The results of a protein–protein interaction (PPI) network analysis indicate that AJS-PDRN reduces cellular oxidative damage by upregulating the expression of intracellular selenoprotein family members. In summary, our findings reveal that AJS-PDRN mitigates H_2_O_2_-induced oxidative damage through multiple pathways, underscoring its significant potential in the prevention and treatment of diseases caused by oxidative stress.

## 1. Introduction

Sea cucumbers, which are classified within the phylum Echinodermata and the class Holothuroidea, have long been recognized as a traditional nutritious food in China and other Asian countries. Currently, more than 1700 species of sea cucumbers have been identified worldwide, with the majority found in temperate and tropical regions. The Asia–Pacific region, in particular, boasts the greatest diversity and abundance of sea cucumber species and resources [1]. In recent years, sea cucumbers have received significant attention as a valuable source for drug development. This is due to their rich composition of unique bioactive substances, including polysaccharides, saponins, lipids, peptides, and others [2]. These active ingredients have important physiological functions in the human body. For example, sea cucumber polysaccharides have various biological benefits such as antitumor [3], antioxidant [4], and anticoagulant activities [5]. Small molecule peptides extracted from sea cucumbers demonstrate antidiabetic [6], enhancing immunity [7], anticancer [8], and antibacterial functions [9]. Sea cucumber saponins have various physiological benefits such as antitumor [10], anti-Parkinson’s disease [11], and immune regulation functions [12]. Therefore, sea cucumbers, as a precious marine food, have great potential in promoting drug research and development.

Under normal physiological conditions, the body has an antioxidant defense system that helps maintain a balance between the generation and elimination of free radicals. However, when this balance is disrupted, excessive free radicals can cause cellular damage, leading to oxidative stress. This oxidative stress can result in dysfunction and lesions within the body [13,14]. Numerous studies have shown that oxidative stress is commonly associated with the pathogenesis of diseases such as aging, diabetes, cancer, ischemia–reperfusion injury, and atherosclerosis [15,16,17]. Therefore, it is crucial to intake sufficient antioxidants to prevent or mitigate oxidative stress induced by free radicals for maintaining normal physiological functions and reducing the occurrence of diseases. However, research has indicated that some commonly used synthetic antioxidants, such as tert-butylhydroquinone (TBHQ), butyl hydroxyanisole (BHA), and propyl gallate (PG), tend to be toxic and carcinogenic [18]. As a result, the search for safe and effective natural antioxidants has become a prominent focus in antioxidant research in recent years.

Polydeoxyribonucleotide is a mixture of nucleotides with molecular weights ranging from 50 to 1500 kDa [19]. Currently, PDRN is primarily derived from the human placenta, salmon sperm, and trout sperm [20]. Research has shown that PDRN is an adenosine A_2A_ receptor agonist, which exerts antioxidant activity by activating the Keap1-Nrf2/HO-1 pathway, thereby increasing the activity of endogenous antioxidant enzymes and promoting the clearance of free radicals [21]. Additionally, PDRN exhibits various biological activities, including anti-inflammatory and anti-apoptotic effects, the promotion of angiogenesis, and the facilitation of tissue repair [22].

Proteomics is a powerful analytical technology that detects the structure, function, and content of all proteins in organisms at the molecular level. It has been widely used in studying the antioxidant mechanisms of various natural active substances [23,24]. To investigate the antioxidant effects of specific active compounds or drugs, RAW264.7 macrophages are often used as an in vitro model. This study evaluated the in vitro antioxidant capacity of AJS-PDRN through DPPH, ABTS, and hydroxyl radical scavenging tests. We used H_2_O_2_ to induce oxidative damage in RAW264.7 cells to establish an in vitro oxidative damage model. By measuring the survival rate and biomarker content of RAW264.7 cells under different treatment conditions, we investigated the protective effect of AJS-PDRN on the oxidative damage model. Additionally, proteomic analysis was performed using isobaric tags for relative and absolute quantification (iTRAQ) to elucidate the specific mechanisms through which AJS-PDRN exerts its protective effect and prevents RAW264.7 cell damage.

## 2. Results

### 2.1. In Vitro Antioxidant Experiment of AJS-PDRN

**Figure 1:**
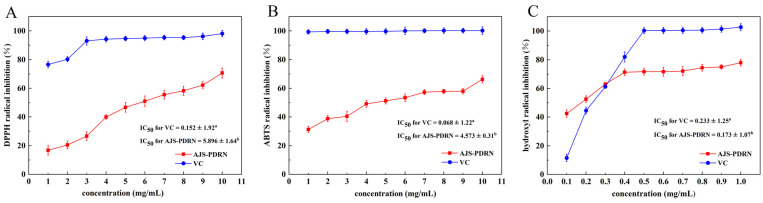
AJS-PDRN scavenging assays for DPPH, ABTS, and hydroxyl radicals. (**A**) AJS-PDRN scavenging assay of DPPH radicals; (**B**) AJS-PDRN scavenging assay of ABTS radicals; and (**C**) AJS-PDRN scavenging assay of hydroxyl radicals. ^a,b^ IC_50_ data with different alphabets show significantly different values (*p* < 0.05).

DPPH, ABTS, and hydroxyl radical scavenging assays were used to assess the antioxidant capacity of AJS-PDRN. The same concentration of vitamin C (VC) was chosen as a control, and the results are shown in Figure 1. 

As the concentration of AJS-PDRN increases, there is a corresponding increase in the scavenging rate of DPPH radicals (Figure 1A). Specifically, at a concentration of 10 mg/mL, the DPPH radical scavenging rate reaches 70.6%, representing a 53.92% increase compared to a concentration of 1 mg/mL. Furthermore, the IC_50_ values of AJS-PDRN and VC for scavenging DPPH radicals are 5.896 ± 1.64 mg/mL and 0.152 ± 1.92 mg/mL, respectively. Similarly, the ability of AJS-PDRN to scavenge ABTS radicals increases with higher concentrations (Figure 1B). At a concentration of 10 mg/mL, the scavenging rate of ABTS radicals by AJS-PDRN is 66.2%, indicating a 35.0% increase compared to 1 mg/mL. The IC_50_ values of AJS-PDRN and VC for scavenging ABTS radicals are 4.573 ± 0.31 mg/mL and 0.068 ± 1.22 mg/mL, respectively. In the hydroxyl radical scavenging assay, a similar trend was observed. As the concentration of AJS-PDRN increased, its ability to scavenge hydroxyl radicals also increased (Figure 1C). The IC_50_ values for hydroxyl radical scavenging by AJS-PDRN and VC were 0.173 ± 1.07 mg/mL and 0.233 ± 1.25 mg/mL, respectively. In general, the lower the IC_50_ value of a natural antioxidant, the stronger its inhibitory activity. Therefore, AJS-PDRN exhibited a stronger inhibitory effect on hydroxyl radicals than VC, indicating a highly potent hydroxyl radical scavenging activity. However, its inhibitory capacity for DPPH and ABTS radicals was moderate. We speculate that this may be related to the chemical structure of AJS-PDRN. DPPH and ABTS are relatively stable radicals, and their scavenging mechanisms primarily rely on the hydrogen-donating ability of the antioxidant [25]. In contrast, hydroxyl radicals are highly reactive and unstable, and scavenging them usually requires antioxidants with high electron-donating capacity and rapid reactivity [26]. Overall, the total antioxidant activity of AJS-PDRN was lower than that of VC. Compared to other identified natural antioxidants, AJS-PDRN demonstrated stronger hydroxyl radical inhibitory activity than the polysaccharide PPP-2 extracted from persimmon peels (IC_50_: 4.08 mg/mL) [27]. Similarly, AJS-PDRN exhibited greater radical scavenging activity compared to PDRN extracted from salmon sperm [28]. In summary, the results of the three assays indicated that within a specific concentration range, the radical scavenging ability of AJS-PDRN showed a dose-dependent increase, demonstrating significant antioxidant activity.

### 2.2. Protective Effect of AJS-PDRN on H_2_O_2_-Induced RAW264.7 Cell Injury Model

#### 2.2.1. Effect of AJS-PDRN on RAW264.7 Cell Viability

**Figure 2:**
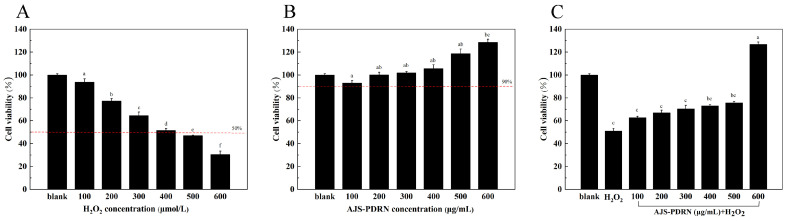
Effects of AJS-PDRN on RAW264.7 cell viability with/without H_2_O_2_ induction. (**A**) Cells treated with the indicated concentrations of H_2_O_2_ for 4 h; (**B**) cells treated with the indicated concentrations of AJS-PDRN for 6 h; and (**C**) cells pretreated with the indicated concentrations of AJS-PDRN for 6 h and then stimulated with 400 μM H_2_O_2_ for 4 h. The results are presented as the mean ± SD of three replicates. Different letters at the top of the columns indicate statistically significant differences.

Hydrogen peroxide can induce various oxidative stresses in cells. By treating cells with exogenous hydrogen peroxide to induce oxidative damage, researchers can investigate the protective effects of active substances on cells. However, higher doses often cause cell death [29]. The cell survival rate is the most intuitive indicator reflecting the degree of cell damage and death caused by external redox imbalances. Therefore, it is a crucial indicator for establishing an oxidative stress model [30]. As shown in Figure 2A, within the concentration range of 100–600 μmol/L, there is a negative correlation between H_2_O_2_ concentration and cell survival rate. At an H_2_O_2_ concentration of 400 μmol/L, the cell survival rate is 52 ± 1.8%, meeting the standard for an oxidative damage model (50–70% cell survival rate). Therefore, 400 μmol/L was chosen as the optimal concentration to induce the damage model.

Following treatment of RAW264.7 cells with varying concentrations (100–600 μg/mL) of AJS-PDRN for 6 h, the survival rate of the cells was above 90%, which indicated that AJS-PDRN was essentially non-toxic to the cells in this range. At a concentration of 200 μg/mL or higher, the survival rate of RAW264.7 cells exceeded 100%, suggesting that AJS-PDRN promotes cell proliferation (Figure 2B).

By determining the effects of different concentrations of AJS-PDRN pretreatment on the viability of RAW264.7 cells induced by H_2_O_2_, we observed that AJS-PDRN had a significant protective effect on damaged cells in the selected concentration range (100–600 μg/mL). As depicted in Figure 2C, following treatment with 400 μmol/L H_2_O_2_, the cell survival rate was 52.0 ± 3.6% of the normal control. Pretreatment of oxidatively damaged RAW264.7 cells with different concentrations of AJS-PDRN significantly increased cell survival. At a concentration of 600 μg/mL, the cell survival rate reached 126.8%. Their results indicate that AJS-PDRN could protect the cells from oxidative damage and thus improve cell viability.

#### 2.2.2. Oxidative Stress Biomarker Levels in RAW264.7 Cells

**Figure 3:**
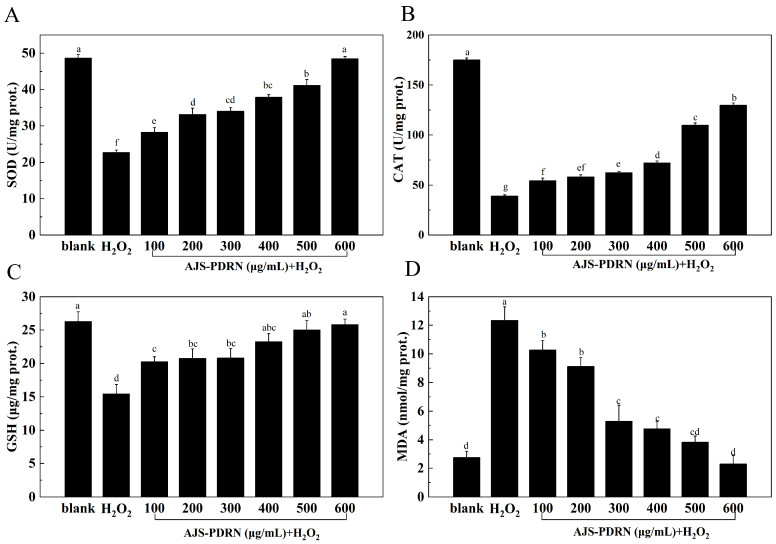
Effects of AJS-PDRN on the levels of oxidative stress biomarkers in H_2_O_2_-induced RAW264.7 cells. (**A**) SOD activity; (**B**) CAT activity; (**C**) GSH content; and (**D**) MDA content. The results are presented as the mean ± SD of three replicates. Different letters at the top of the columns indicate statistically significant differences.

To evaluate the antioxidant effect of AJS-PDRN on H_2_O_2_-induced RAW264.7 cell injury, cells cultured in blank, H_2_O_2_, and AJS-PDRN groups were collected, and the levels of antioxidant indexes, such as superoxide dismutase (SOD), catalase (CAT), glutathione (GSH), and malondialdehyde (MDA), were determined in cells of each group. The results are shown in Figure 3.

SOD is one of the most effective intracellular enzymes that scavenges superoxide anions (O_2_^•−^) free radicals and H_2_O_2_. This enzyme reduces oxidative damage and cytotoxicity, thus alleviating intracellular oxidative stress [31]. As shown in Figure 3A, the SOD content in the H_2_O_2_ group was significantly lower than in the normal group, indicating that H_2_O_2_ damaged RAW264.7 cells and reduced SOD activity. Compared to the H_2_O_2_ group, the intracellular SOD content significantly increased after pretreatment with AJS-PDRN. Additionally, as the concentration of AJS-PDRN increased, the intracellular SOD content proportionally increased. At an AJS-PDRN concentration of 600 μg/mL, the intracellular SOD content increased by 53.18% compared to the H_2_O_2_ group. This indicates that AJS-PDRN can enhance the SOD activity in RAW264.7 cells, protect them from H_2_O_2_ damage, and strengthen the cellular antioxidant system.

CAT maintains the body’s redox balance by scavenging excess free radicals and catalyzing the decomposition of H_2_O_2_ into H_2_O and O_2_ [32]. As shown in Figure 3B, the CAT content in the H_2_O_2_ group was significantly lower than that in the blank group, indicating that H_2_O_2_ damaged RAW264.7 cells and reduced CAT activity. Compared to the H_2_O_2_ group, the intracellular CAT content significantly increased after pretreatment with AJS-PDRN. Moreover, as the concentration of AJS-PDRN increased, the intracellular CAT content also increased. At an AJS-PDRN concentration of 600 μg/mL, the intracellular CAT content increased by 69.77% compared to the H_2_O_2_ group. This demonstrates that AJS-PDRN can enhance the CAT activity in RAW264.7 cells and protect them against H_2_O_2_-induced damage.

GSH is an endogenous antioxidant that effectively eliminates lipid peroxides in the body [33]. As shown in Figure 3C, the GSH content in the H_2_O_2_ group was significantly lower than in the blank group, indicating that H_2_O_2_ damaged RAW264.7 cells and reduced GSH content. Compared to the H_2_O_2_ group, the intracellular GSH content significantly increased after pretreatment with AJS-PDRN. Additionally, as the concentration of AJS-PDRN increased, the intracellular GSH content also increased. At an AJS-PDRN concentration of 600 μg/mL, the intracellular GSH content increased by 38.76% compared to the H_2_O_2_ group. This demonstrates that AJS-PDRN can enhance GSH content in RAW264.7 cells and protect them from H_2_O_2_-induced damage.

MDA is a product of lipid peroxidation caused by oxidative stress. It reacts with biological macromolecules, causing cytotoxicity and serving as an indicator of the body’s peroxidation levels [34]. As shown in Figure 3D, the MDA content in RAW264.7 cells was significantly higher in the H_2_O_2_ group. In comparison to the H_2_O_2_ group, the MDA levels in RAW264.7 cells pretreated with AJS-PDRN were significantly reduced, gradually returning to the level of the normal group with increasing AJS-PDRN concentration. Therefore, AJS-PDRN can reduce the lipid peroxidation caused by H_2_O_2_ and exert antioxidant activity.

### 2.3. Effect of AJS-PDRN on Protein Expression Profiles in RAW264.7 Cells Induced by H_2_O_2_

#### 2.3.1. Screening of Differentially Expressed Proteins

**Figure 4:**
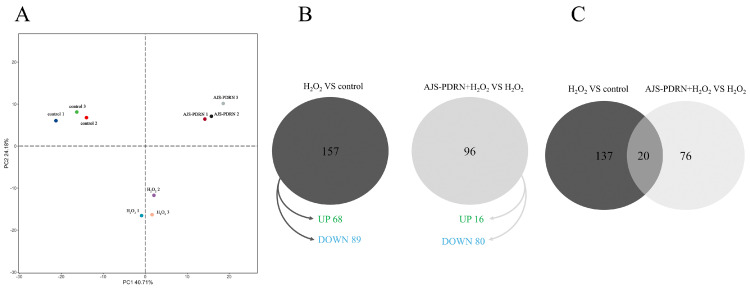
Effect of AJS-PDRN on H_2_O_2_-induced protein expression profile in RAW264.7 cells. (**A**) PCA based on quantitative data of the selected proteins in each group (*n* = 3 for each group); (**B**) Venn diagram of DEPs in the H_2_O_2_ group and the AJS-PDRN group; and (**C**) Venn diagram demonstrating the number of DEPs identified in each comparison.

To investigate the protective effects of AJS-PDRN against oxidative stress-induced damage in RAW264.7 cells, we conducted a proteomic analysis utilizing iTRAQ tag quantification technology. Samples from three replicates of each subgroup (normal, H_2_O_2_, and AJS-PDRN-H_2_O_2_) were pooled and subjected to mass spectrometry for protein isolation. Leveraging the mouse (mmu) protein database, we identified a total of 7631 proteins (1% FDR and protein-level FDR). Principal component analysis (PCA) was performed on the expression levels of reliable proteins. The results showed a high level of quantitative repeatability in the degree of aggregation among repeated samples, with significant differences observed between various samples (Figure 4A). Proteins meeting the criteria of having at least one peptide, a fold change of ≥1.5 or ≤0.67, and a *p*-value < 0.05 were considered differentially expressed proteins (DEPs).

To identify the proteins whose expression was influenced by AJS-PDRN, we analyzed the DEPs that were either increased or decreased in both the H_2_O_2_ group and the AJS-PDRN-H_2_O_2_ group using Venn diagram analysis (Figure 4B). Our results showed that the H_2_O_2_ group had 157 DEPs compared to the normal group, with 68 upregulated and 89 downregulated. The AJS-PDRN-H_2_O_2_ group had 96 DEPs compared to the H_2_O_2_ group, of which 16 were upregulated and 80 were downregulated. Intriguingly, among the 20 DEPs screened in both comparisons, six were upregulated in the H_2_O_2_ group and downregulated in the AJS-PDRN-H_2_O_2_ group. Only one DEP was downregulated in the H_2_O_2_ group and upregulated in the AJS-PDRN-H_2_O_2_ group (Figure 4C, Table 1).

#### 2.3.2. Gene Ontology (GO) Functional Annotation Enrichment Analysis of DEPs

**Figure 5:**
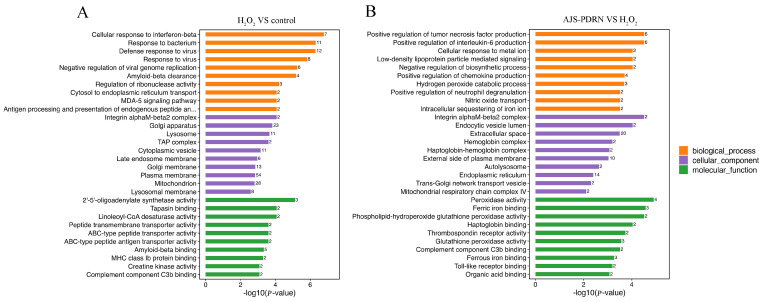
GO enrichment analysis of DEPs. (**A**) The H_2_O_2_ group vs. the control group; (**B**) the AJS-PDRN group vs. the H_2_O_2_ group. The number after each term represents the number of differential proteins annotated to that term.

In order to comprehensively understand the functional classification of the DEPs, we used DAVID bioinformatics resource 6.8 to conduct GO functional annotation analysis of the DEPs. Enrichment analysis was performed from the aspects of biological process (BP), cellular component (CC), and molecular function (MF). The top 10 terms that were significantly enriched (*p* < 0.05) in each category are listed in Figure 5. In the comparison between the control and H_2_O_2_ groups (Figure 5A), GO BP analysis revealed that the DEPs induced by H_2_O_2_ were enriched in processes such as cellular response to interferon-beta, response to the bacterium, defense response to the virus, and response to the virus. Under oxidative stress conditions, cells activate a series of defense mechanisms, including the enhancement of interferon-β-related antiviral responses and immune responses against bacteria. GO CC analysis showed that these proteins are mainly involved in CCs such as the Golgi apparatus, lysosome, plasma membrane, and mitochondrion. These alterations in protein levels suggest that oxidative stress impacts multiple critical cellular sites, encompassing protein processing and degradation (Golgi apparatus and lysosome), cell signaling and material exchange (plasma membrane), and energy metabolism (mitochondria). GO MF analysis revealed that the MFs primarily involved in this group of differential proteins include 2′-5′-oligoadenylate synthetase activity, tapasin binding, linoleoyl-CoA desaturase activity, peptide transmembrane transporter activity, etc. These functions are associated with antiviral responses, antigen presentation, fatty acid metabolism, and transmembrane substance transport, which indicate that cells maintain function and survival through various pathways in response to oxidative stress.

In the comparative analysis of the AJS-PDRN and H_2_O_2_ groups (Figure 5B), GO BP analysis revealed that the differentially expressed proteins between the AJS-PDRN and H_2_O_2_ groups were primarily enriched in the processes of positive regulation of tumor necrosis factor production, positive regulation of interleukin-6 production, and positive regulation of chemokine production, as well as the hydrogen peroxide catabolic process. The upregulation of tumor necrosis factor, interleukin-6, and the production of chemokines suggests that AJS-PDRN may alleviate oxidative stress-induced damage by modulating these key inflammatory mediators. Moreover, the enrichment of hydrogen peroxide decomposition processes indicates that AJS-PDRN may mitigate oxidative damage by enhancing the cells’ capacity to eliminate H_2_O_2_. GO CC analysis showed that these differential proteins were primarily enriched in CCs such as integrin alphaM-beta2 complex, extracellular space, external side of the plasma membrane, and endoplasmic reticulum. Research indicates that proteins within integrin complexes and on the extracellular side of the plasma membrane typically participate in cell adhesion and signal transduction processes [35]. This suggests that AJS-PDRN likely exerts its protective effects by enhancing intercellular interactions and signal transduction. Moreover, the enrichment of proteins in the endoplasmic reticulum suggests that AJS-PDRN may alleviate endoplasmic reticulum stress by modulating protein synthesis and folding. GO MF analysis showed that the differentially expressed proteins in this group were primarily involved in MFs, including peroxidase activity, ferric iron binding, glutathione peroxidase activity, and ferrous iron binding. These MFs indicate that AJS-PDRN exerts its protective effects primarily by enhancing the antioxidant capacity of cells. In summary, AJS-PDRN safeguards RAW264.7 cells from H_2_O_2_-induced oxidative damage through multiple mechanisms. AJS-PDRN not only regulates the production of inflammatory mediators and enhances anti-inflammatory responses but also exerts its protective effects by boosting the cells’ antioxidant capacity and modulating the extracellular matrix and signal transduction pathways.

#### 2.3.3. KEGG Pathway Enrichment Analysis of DEPs

**Figure 6:**
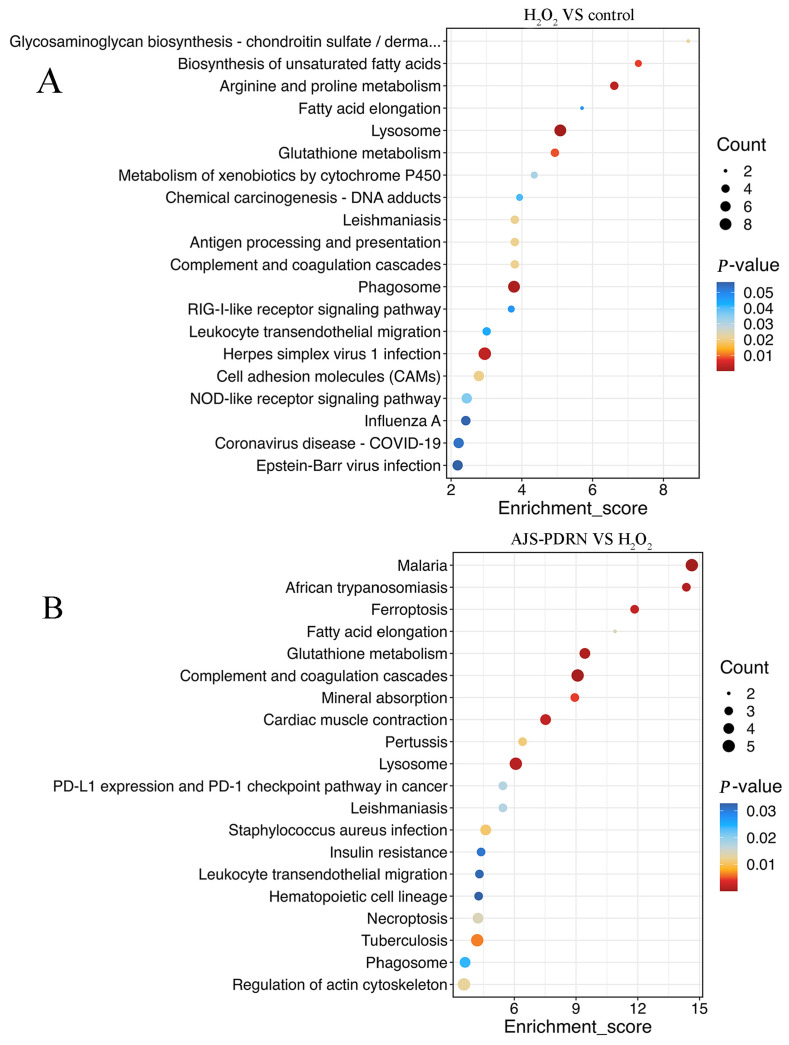
The bubble diagram of KEGG pathway analysis of DEPs. (**A**) The H_2_O_2_ group vs. the control group; (**B**) the AJS-PDRN group vs. the H_2_O_2_ group. The larger the bubble the greater the number of differential proteins contained in the entry. The color of the bubbles changes from blue to red, indicating that the smaller the enrichment *p*-value, the greater the degree of significance.

In order to further analyze the mechanism of action of AJS-PDRN, the KEGG database was used to perform enrichment analysis on the selected metabolic pathways of DEPs, screening for enriched metabolic terms with a *p*-value of less than 0.05. The results demonstrated that the metabolic pathways that exhibited significant enrichment for the differential proteins in the H_2_O_2_ and control groups included arginine and proline metabolism, biosynthesis of unsaturated fatty acids, glutathione metabolism, and NOD-like receptor signaling pathway (Figure 6A). The accumulation of these DEPs in the aforementioned metabolic pathways illustrates the cell’s multifaceted response to H_2_O_2_-induced oxidative stress. These pathways encompass the regulation of metabolism, the repair of damaged cell membranes, the elimination of reactive oxygen species, and the activation of immune and inflammatory responses. The synergistic action of these mechanisms aids cells in maintaining survival and function in response to oxidative damage. In the comparison between the AJS-PDRN group and the H_2_O_2_ group, the signaling pathways enriched by differential proteins were primarily glutathione metabolism, fatty acid elongation, complement and coagulation cascades, and lysosome (Figure 6B). Studies suggest that fatty acid elongation plays a critical role in the construction and repair of cell membranes [36]. Oxidative stress may damage cell membranes, leading to impaired cellular function. Therefore, AJS-PDRN might improve cell survival under oxidative stress by promoting fatty acid elongation and assisting in the repair of damaged membrane structures. Furthermore, the complement and coagulation cascades are crucial defense mechanisms in the body, involved in immune and inflammatory responses [37]. In summary, AJS-PDRN mitigates H_2_O_2_-induced oxidative damage through multiple pathways. The enrichment results of DEPs in the above pathways suggest that AJS-PDRN protects cells from oxidative damage by enhancing antioxidant capacity, promoting membrane repair, regulating immune and inflammatory responses, and augmenting intracellular degradation functions. 

#### 2.3.4. Analysis of Protein Interaction Network

**Figure 7:**
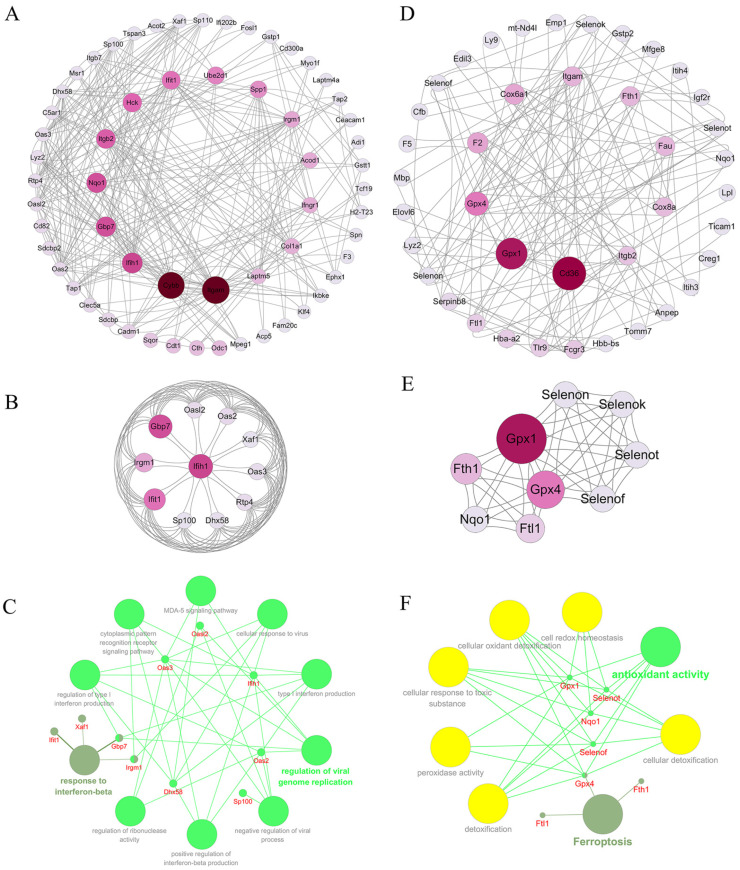
PPI network analysis of DEPs. (**A**) PPI network diagram of all DEPs with interactions in the H_2_O_2_ group vs. the control group. (**B**) The highest-scoring sub-network in the H_2_O_2_ group was selected using the MCODE algorithm. (**C**) The major GO BP terms were significantly enriched by 11 proteins in the sub-network, as well as the related DEPs. (**D**) PPI network diagram of all DEPs with interactions in the AJS-PDRN group vs. the H_2_O_2_ group. (**E**) The highest-scoring sub-network in the AJS-PDRN group was selected using the MCODE algorithm. (**F**) The major GO BP terms and KEGG pathway were significantly enriched by 9 proteins in the sub-network, as well as the related DEPs.

Research has shown that most proteins do not operate independently in organisms, but rather need to interact with other proteins in order to fulfill their functions [38]. Consequently, we conducted a network analysis of the two groups of differential proteins using a protein–protein interaction network. The PPI network of differential proteins between two different groups was constructed using the STRING database and Cytoscape software (version 3.10.2). In the comparison of the H_2_O_2_ group with the control group, the differential protein PPI network contained 59 nodes and 298 interactions. Each node was ranked according to the Betweenness Centrality (BC) using the Cytoscape plugin CytoNCA. The proteins with more interactions are displayed in the inner circle of the PPI network map (Figure 7A). Furthermore, we also used molecular complex detection (MCODE) algorithm analysis for DEPs to establish PPI networks. MCODE is a density-based non-overlapping clustering algorithm that utilizes seed nodes as the center to expand to neighboring nodes, which are then screened to construct complexes in a protein–protein interaction network. This approach enables the identification of potential biological mechanisms related to the network [39]. We screened the top cluster with the highest clustering score (score: 9.4), and the results are shown in Figure 7B. This cluster comprises a total of 11 protein nodes and 94 interactions. To gain further understanding of the function of these proteins, we performed biological process enrichment and KEGG pathway analysis using ClueGO and CluePedia, and the result is shown in Figure 7C. The proteins are primarily engaged in biological processes such as the regulation of viral genome replication (Gbp7, Ifih1, Oas2, Oas3, and Oasl2) and the response to interferon-beta (Gbp7, Ifih1, Irgm1, and Xaf1). 

In the comparison of the AJS-PDRN group and the H_2_O_2_ group, the differential protein PPI network contained a total of 40 protein nodes and 160 interactions (Figure 7D). Additionally, the module with the highest clustering score, identified using the MCODE algorithm, is shown in Figure 7E. This cluster comprises nine protein nodes and 48 interactions, most of which are members of the selenoprotein family. It is well known that selenium is an essential trace element for the human body, with strong antioxidant properties. The physiological functions of selenium are primarily realized through selenoproteins [40]. Numerous studies have demonstrated that selenoproteins play a crucial role in regulating the body’s redox balance [41,42]. Furthermore, the results of ClueGO and CluePedia enrichment analysis are shown in Figure 7F. The GO biological processes involving these proteins are all related to antioxidant activity (Gpx1, Gpx4, Nqo1, Selenot, and Selenof), and the only associated KEGG signaling pathway is Ferroptosis (Gpx4, Fth1, and Ftl1).

## 3. Discussion

As a traditional health food, sea cucumber has high nutritional and medicinal value. The main edible part of the sea cucumber is its body wall; therefore, most research focuses on this component [43]. During sea cucumber processing, viscera byproducts, such as sea cucumber sperm, are often underutilized. Studies have shown that sea cucumber viscera are rich in taurine, polydeoxyribonucleotides, arginine, unsaturated fatty acids, trace elements, and other active compounds, in addition to those similar to the body wall [44,45]. Therefore, sea cucumber viscera also possess various biological activities, including antioxidant, antitumor, anticancer, and immunomodulatory effects [46,47]. The lack of awareness and neglect of the active substances in sea cucumber viscera lead to significant resource wastage and loss. Additionally, research on the active substances of sea cucumber viscera, both domestically and internationally, remains limited, and few relevant products have been developed. Therefore, sea cucumber sperm was chosen as the raw material for this study to extract PDRN, which exhibits various biological activities.

It is well known that H_2_O_2_ is highly susceptible to diffusion through cell membranes, reaching nuclear tissues and initiating a series of oxidative stress reactions. Moreover, H_2_O_2_ is readily obtainable and relatively stable chemically. Therefore, exogenous H_2_O_2_ has become an important tool for studying cellular oxidative damage [29]. This is of great significance for researchers to study the cytoprotective and reparative roles of bioactive substances. Studies have shown that oxidative stress is usually associated with the development of various diseases, and the excess reactive oxygen species it induces can cause extensive damage to DNA and cellular macromolecules, such as altering membrane fluidity, destroying the cytoskeleton, and promoting protein denaturation, etc., which ultimately causes a series of cellular dysfunction phenomena such as apoptosis and cancer [17]. Therefore, the search for natural active substances with high antioxidant properties is crucial to protect the body cells from oxidative stress damage.

PDRN is a naturally occurring low-molecular-weight DNA derivative extracted and prepared primarily from the sperm cells of trout or salmon [20]. PDRN is commonly used as an adenosine A_2A_ receptor activator, which exerts various physiological effects [48]. Currently, most studies on PDRN activity have focused on anti-inflammatory, anti-apoptotic, and tissue repair properties [22]. However, there are relatively few studies on its antioxidant activity. In this study, we report for the first time that PDRN was extracted from *Apostichopus japonicus* sperm, and its antioxidant activity was investigated using DPPH, ABTS, and hydroxyl radical scavenging assays. The results of all three assays showed that AJS-PDRN exhibits excellent antioxidant properties.

The excellent antioxidant activity of AJS-PDRN suggests that AJS-PDRN may have the ability to protect cells from oxidative stress damage. Therefore, in this study, we successfully constructed a cell injury model using exogenous H_2_O_2_ to induce oxidative damage in RAW264.7 cells. The cytoprotective and reparative abilities of AJS-PDRN were evaluated in vitro. The results showed that AJS-PDRN had superior protective abilities, as demonstrated by its promotion of cell proliferation and reduction in H_2_O_2_-induced cellular damage (Figure 2B,C).

To maintain the balance of the body’s redox system, the body relies on its antioxidant enzyme system, which consists of key enzymes such as SOD and CAT [49]. These enzymes protect cells from oxidative damage by scavenging excess ROS and free radicals from the cells. SOD is the enzyme that catalyzes the disproportionation of superoxide anion radicals to produce hydrogen peroxide and oxygen [31]. CAT is the antioxidant enzyme that is present in virtually all organisms and catalyzes the conversion of hydrogen peroxide to water and oxygen [32]. GSH is an important antioxidant that can scavenge lipid peroxides through Gpx4 and inhibit Ferroptosis [33]. MDA is a byproduct of lipid peroxidation and serves as a marker of oxidative stress [34]. In this study, we evaluated the reparative effect of AJS-PDRN on cellular damage by measuring the levels of these four biomarkers in RAW264.7 cells under different treatment conditions. The results showed that AJS-PDRN significantly increased the intracellular levels of SOD, CAT, and GSH, and decreased MDA content, attenuating H_2_O_2_-induced RAW264.7 cell injury in a dose-dependent manner. Currently, commonly used natural antioxidants also encompass polyphenolic compounds. Phenolic compounds extracted from fruit wines can significantly enhance the activity of antioxidant enzymes in cells and mitigate the levels of MDA in cells subjected to experimentally induced oxidative stress [50]. In conclusion, AJS-PDRN demonstrates potent antioxidant activity and exhibits superior protective and reparative effects on H_2_O_2_-induced RAW264.7 cell injury.

In order to elucidate the mechanism of the protective effect of AJS-PDRN on H_2_O_2_-induced RAW264.7 cells, DEPs in the cells were identified by the iTRAQ proteomics method in this study. The results showed that a total of 157 DEPs were identified in the H_2_O_2_ group versus the blank group, while 96 DEPs were identified in the AJS-PDRN group and the H_2_O_2_ group. Interestingly, six DEPs (Hba, Nqo1, Maf1, Myl2, Kdm8, and Arhgef39) were found to have upregulated expression in the H_2_O_2_ group, whereas their expression was downregulated after pretreatment with AJS-PDRN. Additionally, one DEP (Cox8a) exhibited the opposite expression pattern (Table 1).

Hemoglobin subunit alpha (Hba) is a crucial component of hemoglobin that is primarily responsible for oxygen transport. In addition, hemoglobin is involved in the detoxification of cellular oxidants and protects cells from oxidative damage. Studies have shown that hemoglobin not only transports oxygen in red blood cells but also has antioxidant functions in other cells. Hemoglobin can reduce oxidative damage by interacting with peroxides via its subunit α and reducing the production of free radicals [51]. This protective mechanism may explain the upregulation of hemoglobin subunit α expression in the H_2_O_2_ group after induction by H_2_O_2_. Nqo1 is a key antioxidant enzyme that reduces quinones to less toxic hydroquinones, thus reducing the generation of reactive oxygen species and decreasing oxidative stress-induced cellular damage [52]. Studies have shown that Nqo1 expression is regulated by the Nrf2 (nuclear factor E2-related factor 2)/ARE (antioxidant response element) signaling pathway. Under oxidative stress conditions, Nrf2 is released from the Keap1 complex, translocates to the nucleus, and binds to ARE, initiating the expression of various antioxidant genes such as Nqo1 [53]. Therefore, the increased expression of the Nqo1 protein in the H_2_O_2_ group is a self-protective mechanism of cells. Maf1 is a major repressor of RNA polymerase III. Under oxidative stress conditions, Maf1 reduces the metabolic burden of cells by inhibiting the transcriptional activity of RNA polymerase III, thus reducing oxidative damage [54]. Upregulation of Maf1 protein was detected in the H_2_O_2_ group, suggesting that cells may reduce oxidative damage by decreasing metabolic burden. Myl2 is a member of the myosin light chain family that is expressed mainly in cardiac and skeletal muscle and is involved in the regulation of muscle contraction [55]. Myl2 regulates the contraction and relaxation of muscle fibers by binding to myosin-heavy chains in cardiac and skeletal muscle. Upregulation of Myl2 expression after treatment with H_2_O_2_ enhances cellular resistance to oxidative damage, maintains cytoskeletal stability and function, and may promote cell survival by regulating oxidative stress-associated signaling pathways, such as the Nrf2 pathway. Kdm8 is a histone demethylase that can help cells repair DNA damage and maintain gene stability by regulating chromatin structure and gene expression [56]. In the H_2_O_2_ group, upregulation of Kdm8 expression may enhance the antioxidant capacity and DNA repair mechanism of cells, reducing oxidative stress-induced damage. The reduction in Kdm8 protein levels after pretreatment with AJS-PDRN indicates its potential repair role. Arhgef39 is a guanine nucleotide exchange factor belonging to the Rho GTPase-regulated protein family, affecting various cellular processes [57]. Oxidative stress activates a variety of signaling pathways, including the Rho family of GTPase signaling pathways. Arhgef39, through the activation of Rho GTPases, may regulate cell proliferation, survival, and migration during cellular response to oxidative stress.

Cox8a is a subunit of the mitochondrial respiratory chain complex IV (cytochrome c oxidase) involved in electron transfer and ATP synthesis [58]. Increased Cox8a expression may improve mitochondrial electron transfer efficiency, thereby reducing reactive oxygen species generation under oxidative stress and enhancing cell survival.

Regarding the analysis of GO and KEGG pathways, the present study revealed that AJS-PDRN significantly affects the positive regulation of tumor necrosis factor production, positive regulation of interleu-kin-6 production, hydrogen peroxide catabolic process, extracellular space, endoplasmic reticulum, peroxidase activity, and glutathione peroxidase activity, among other related GO terms (Figure 5B). These GO terms are closely related to the antioxidant function as well as the anti-inflammatory activity of the organism.

The KEGG signaling pathways enriched in the AJS-PDRN group compared with the H_2_O_2_ group mainly included glutathione metabolism, fatty acid elongation, complement and coagulation cascades, and lysosome. Glutathione is an important intracellular antioxidant that effectively scavenges free radicals and other reactive oxygen species [59]. Therefore, glutathione plays an important role in protecting cells from oxidative damage. The fatty acid extension process plays a key role in cell membrane synthesis and energy metabolism [36]. This suggests a possible role for AJS-PDRN in repairing and maintaining the integrity of membrane structure. In addition, studies have shown that the complement system and coagulation system have important roles in immune defense and damage repair processes in vivo [37]. The enrichment results of the AJS-PDRN pretreated group implied that AJS-PDRN might also have immunomodulatory and anti-inflammatory roles, which need to be further investigated in depth.

In order to further investigate the protection mechanism of AJS-PDRN on the RAW264.7 cell injury model, this study utilized the PPI network to analyze the two groups of DEPs in the experiment separately. Using the MCODE algorithm, we filtered out a sub-network with the highest clustering score from the AJS-PDRN group. The results showed that there were nine protein nodes and 48 interactions in this module. Six of these proteins (Gpx1, Gpx4, Selenon, Selenok, Selenot, and Selenof) are selenoprotein family members. Studies have shown that selenoproteins play an important role in regulating redox homeostasis in the human body [41]. The main mechanism involves the direct scavenging of hydrogen peroxide and organic peroxides through the catalysis of enzymes such as glutathione peroxidase, thioredoxin reductase, and selenoprotein P, which, in turn, attenuates oxidative damage [60]. Subsequently, we enriched and analyzed these nine proteins using ClueGO and CluePedia plugins. The results showed that these proteins were mainly involved in biological processes related to antioxidant activity. In addition, Fth1, Ftl1, and Gpx4 were enriched in the Ferroptosis KEGG signaling pathway. The studies showed that Ferroptosis is a novel mode of non-apoptotic cell death characterized by iron-dependent accumulation of lipid peroxides and altered redox homeostasis [61]. In summary, AJS-PDRN may attenuate cellular oxidative damage by promoting the expression of intracellular selenoprotein family members, enhancing the activity of various intracellular antioxidant enzymes. The proteomic analysis provided initial insights into the in vivo mechanism of action of AJS-PDRN, paving the way for comprehensive studies on its prospective application in oxidative stress-related conditions.

## 4. Materials and Methods

### 4.1. Materials

RAW264.7 cells were purchased from the Chinese Academy of Sciences Cell Bank. Fresh spermary of sea cucumber (*Apostichopus japonicus*) was purchased from Shanshui seafood Co., Ltd. (Yantai, China). Proteinase K (enzyme activity 43.6 U/mg), Spark 2000PDRN Marker, Spark 6× PDRN Loading Buffer, and Spark 5× TBE Buffer were purchased from Shandong SparkJade Biotechnology Co. (Jinan, China); Gelred nucleic acid dye was purchased from Shanghai Sangon Biotech Co. (Shanghai, China); Cell Counting Kit-8 (CCK-8) kit and phenylmethylsulfonyl fluoride (PMSF) were purchased from Shanghai Beyotime Biotechnology Co. (Shanghai, China); the SOD, CAT, MDA and GSH assay kits were all acquired from Beijing Solarbio Biotechnology Co. (Beijing, China); and other chemical reagents were purchased from Sinopharm (Beijing, China).

### 4.2. Extraction and Preparation of AJS-PDRN

Referring to the method of Cawthorn et al. [62], a 0.06 g sample of fresh sea cucumber sperm was placed into a 1.5 mL centrifuge tube using sterilized scissors. A volume of 0.3 mL of lysis buffer (10 mM Tris-HCl, pH 8.0; 2 mM EDTA, pH 8.0; 0.4 M NaCl) was added, followed by the addition of 10% SDS and 0.8 μL of proteinase K. The mixture was incubated in a 50 °C water bath for 12 h for enzymatic digestion. After digestion, the mixture was centrifuged at 10,000 r/min for 1 h. The supernatant was transferred to a new centrifuge tube and 0.5 volumes of 6 M NaCl along with an equal volume of phenol:chloroform:alcohol (25:24:1) were added and mixed thoroughly. After thorough mixing, the mixture was centrifuged at 10,000 r/min for 1 h and the supernatant was transferred to a new centrifuge tube. Subsequently, 0.6 volumes of isopropanol were added to precipitate the DNA, which was then washed twice with 70% (*v*/*v*) ethanol. Finally, the DNA precipitate was dried in an oven at 65 °C and stored at −20 °C for future use.

### 4.3. DPPH Radical Scavenging Assay by AJS-PDRN

The DPPH radical scavenging activity of AJS-PDRN was measured by referring to the method of Shao et al. with slight modifications [63]. In brief, 1 mL of a 0.2 mmol/L DPPH–ethanol solution was combined with 1 mL of sample solutions of varying concentrations, followed by a 30 min reaction at room temperature in the dark. The absorbance value at 517 nm was measured and recorded as A_1_. Subsequently, 1 mL of both the sample solution and anhydrous ethanol were thoroughly mixed and reacted in the dark at room temperature for 30 min, with the absorbance measured as A_2_. Lastly, 1 mL of distilled water was substituted for the sample solution and mixed with 1 mL of DPPH–ethanol solution, allowing the reaction to proceed in the dark for 30 min. The absorbance was then measured, resulting in A_3_. VC was used as a standard solution in a control experiment. The DPPH scavenging effect was calculated as follows:

$$\mathrm{DPPH}\;\mathrm{scavenging}\;\mathrm{rate}\;(\%)=[\frac{1-(A_{1}-A_{2})}{A_{3}}]\times 100\%.$$

### 4.4. ABTS Radical Scavenging Assay by AJS-PDRN

The ABTS free radical scavenging assay was also employed to assess the antioxidant activity of AJS-PDRN [64]. The working solution was prepared by combining an ABTS solution (7 mmol/L) with a potassium persulfate solution (2.45 mmol/L) in equal volumes and shielded from light for a period of 12 h. The absorbance at 734 nm was 0.700 ± 0.005 after dilution with ethanol. The diluted ABTS working solution (4 mL) was mixed with 100 μL of the sample solution and reacted for 10 min under the protection of light. The absorbance at 734 nm was designated as A_1_, and the value of the absorbance measured by using the ethanol solution in lieu of the ABTS working solution was recorded as A_2_. The absorbance measured by reacting a diluted ABTS working solution (4 mL) with 100 μL of an ethanol solution was recorded as A_3_. A control experiment was conducted using VC. The ABTS scavenging effect was calculated as follows:

$$\mathrm{ABTS}\;\mathrm{scavenging}\;\mathrm{rate}\;(\%)=[\frac{1-(A_{1}-A_{2})}{A_{3}}]\times 100\%.$$

### 4.5. Hydroxyl Radical Scavenging Activity

The hydroxyl radical scavenging activity of AJS-PDRN was determined by the method of Souza et al. with slight modifications [65]. An amount of 1 mL of 9 mmol/L FeSO_4_, 9 mmol/L of salicylic acid–ethanol solution, 8.8 mmol/L of hydrogen peroxide solution, and the sample solution were added to the test tube, respectively, and allowed to react for 30 min at 37 °C. The absorbance was measured at 510 nm and counted as A_1_. The salicylic acid was replaced with distilled water, and the absorbance was counted as A_2_. The sample was replaced with distilled water, and the absorbance was counted as A_3_. VC was used as a control experiment. The hydroxyl radical scavenging effect was calculated as follows:

$$•\mathrm{OH}\;\mathrm{scavenging}\;\mathrm{rate}\;(\%)=[\frac{1-(A_{1}-A_{2})}{A_{3}}]\times 100\%.$$

### 4.6. Cell Culture 

The RAW264.7 cells were cultured in Dulbecco’s Modified Eagle Medium (DEME) supplemented with 10% fetal bovine serum (FBS) and 1% dual-antibody reagent (consisting of 100 U/mL of penicillin and 100 mg/mL of streptomycin). The cells were cultured in a humidified environment at 5% CO_2_ at 37 °C, with the medium being changed every two or three days. 

### 4.7. Cell Viability Assay

The viability of the cells was determined using the Cell Counting Kit-8 (CCK-8) method. In brief, RAW264.7 cells (10,000 cells/well) were inoculated into 96-well plates, and then CCK-8 solution (10 µL per well) was added at 0, 24, 48, and 72 h, respectively. The cells were incubated at 37 °C for 2 h, and then the absorbance was measured at 450 nm using an enzyme-labeled instrument (ThermoFisher Scientific, Shanghai, China).

### 4.8. Establishment of H_2_O_2_-Induced Oxidative Stress Model of RAW264.7 Cells

RAW264.7 cells (10,000 cells/well) were seeded in 96-well plates and incubated for 24 h. Following this incubation period, 100 µL of varying concentrations of H_2_O_2_ solution (100 µM, 200 µM, 300 µM, 400 µM, 500 µM, and 600 µM) were added and incubated for an additional 4 h. Subsequently, 10 µL of CCK-8 solution was added, and the cells were further incubated for 1 h. The absorbance was measured at a wavelength of 450 nm and cell viability was calculated.

### 4.9. Toxic Effect of AJS-PDRN on RAW264.7 Cells

RAW264.7 cells (10,000 cells/well) were seeded in a 96-well plate and cultured for 24 h. Then, 100 μL of AJS-PDRN solution of different concentrations (100 µM, 200 µM, 300 µM, 400 µM, 500 µM, and 600 µM) was added, and after 6 h of action, 10 μL of CCK-8 solution was added and incubated for 1 h. The absorbance was measured at a wavelength of 450 nm and cell viability was calculated.

### 4.10. Protective Effect of AJS-PDRN on H_2_O_2_-Induced Cellular Oxidative Stress Model

In order to explore the protective effect of AJS-PDRN on cells, RAW264.7 cells (10,000 cells/well) were seeded in a 96-well plate and cultured for 24 h. The RAW264.7 cells were then treated with AJS-PDRN solution of different concentrations (100 µM, 200 µM, 300 µM, 400 µM, 500 µM, and 600 µM) for 6 h. Following two washes with PBS, the cells were then treated with a 400 μM H_2_O_2_ solution (IC_50_ concentration) for 4 h. Cell viability was assessed using the CCK-8 method.

### 4.11. CAT, SOD, GSH, and MDA in RAW264.7 Cells

RAW264.7 cells (10,000 cells/well) were seeded in a 96-well plate and cultured for 24 h, and a blank group, a control group, and an experimental group were set. The blank group consisted of normally growing cells; cells treated with 2 mL of 400 μM H_2_O_2_ solution served as the control group; and the experimental group added 2 mL of AJS-PDRN of different concentrations to normal cells and cultured them for 6 h, and then added 2 mL of 400 μM H_2_O_2_ solution for 4 h. Finally, according to the instructions of the CAT, SOD, GSH and MDA detection kits, the contents of CAT, SOD, GSH and MDA were measured on the 96-well plate.

### 4.12. Protein Preparation and iTRAQ Labelling

RAW264.7 cells were initially seeded in culture dishes at a density of 2 × 10^4^ cells/cm^2^ and incubated until reaching 80–90% confluence for subsequent iTRAQ-based proteomics analysis. Following the assessment of cell viability and biochemical assays, a high concentration of AJS-PDRN was chosen for the proteomic study. The RAW264.7 cells were then divided into nine groups, comprising three control groups, three H_2_O_2_ groups, and three AJS-PDRN+H_2_O_2_ groups. Cells in the control group underwent normal culture conditions, those in the H_2_O_2_ group were treated to 400 μM H_2_O_2_ for 6 h, and cells in the AJS-PDRN group were pretreated with 600 μM AJS-PDRN for 6 h before being treated with 400 μM H_2_O_2_ for an additional 6 h. Subsequently, cellular proteins were extracted using a protein extraction kit, and protein concentration was determined using a BCA protein assay kit (Solarbio, Beijing, China). All steps were performed in accordance with the manufacturer’s instructions.

RAW264.7 cells in each group were collected by centrifugation and washed twice with PBS. The samples were sonicated three times in lysis buffer (8 M urea, 1% protease inhibitor cocktail) on ice using an ultrasonic cell crusher (Scientz, Shenzhen, China), and the supernatants were collected. Then, trypsin was added to the protein solution at a mass ratio of protein to the enzyme of 30:1, and enzymatic hydrolysis was carried out at 37 °C for 4 h. Subsequently, 2 μL of 0.2% trifluoroacetic acid was immediately added to terminate the enzymatic hydrolysis. Desalting was performed via solid-phase extraction cartridge Sep-Pak C18, followed by drying under vacuum. The digested peptides were then labeled with iTRAQ using the 8-plex kit.

### 4.13. LC-MS/MS High-Resolution Mass Spectrometry Analysis

The labeled peptides were separated using high-pH reversed-phase high-performance liquid chromatography. The trypsin-digested peptides were then analyzed by LC-MS/MS using a timsTOF Pro mass spectrometer (Bruker Daltonics, Bremen, Germany). The mass spectrometry data were analyzed and processed using Spectronaut Pulsar 18.4 software (Biognosys, Zurich, Switzerland). The chromatographic column is an Eksigent C18 (75 μm × 250 mm, 1.7 μm), with mobile phase A being a 0.1% formaldehyde aqueous solution (*v*/*v*), and mobile phase B being a 0.1% formaldehyde in acetonitrile solution. The elution gradient was as follows: 0 min, 5% B; 48 min, 22% B; 53 min, 35% B; 56 min, 90% B; 57 min, 3% B; 60 min, 3% B; and the flow rate was 300 nL/min. Additionally, the external electrospray voltage is 1.5 kV, and the *m*/*z* scanning range of the full scan is from 100 to 1700. 

### 4.14. Protein Identification and Bioinformatic Analysis

The mass spectrometry data were processed using Spectronaut Pulsar 18.4 software. Proteins possessing at least one unique peptide with an unused value of more than 2 were screened for further identification. Differentially expressed proteins were screened with a *p*-value < 0.05 and fold change ≥ 1.5 or fold change ≤ 0.67. Additionally, functional classification and annotation were performed using DAVID Bioinformatics Resource 6.8 with GO to determine enrichment in cellular components, biological processes, and molecular function. Pathway analysis was conducted using the KEGG database to determine enrichment in signaling pathways. Protein interaction analysis was carried out using the STRING database and Cytoscape software.

### 4.15. Statistical Processing and Analysis

The physiological indicators or proteomic analysis for all samples were repeated three times, and the results are presented as mean ± standard deviation (SD). Statistical analysis of the data was conducted using SPSS Statistics 26 software, and Duncan’s multiple test was performed via one-way analysis of variance (*p* < 0.05 indicating significant differences).

## 5. Conclusions

In this study, we prepared PDRN from the sperm of *Apostichopus japonicus* for the first time and evaluated the antioxidant activity of AJS-PDRN through DPPH, ABTS, and hydroxyl radical scavenging assays. In subsequent in vitro antioxidant assays, we found that AJS-PDRN pretreatment significantly increased the survival of H_2_O_2_-induced RAW264.7 cells. The altered levels of oxidative stress biomarkers indicated that AJS-PDRN possesses significant antioxidant activity. Furthermore, iTRAQ proteomics analysis reveals that the protective effects of AJS-PDRN on the cell injury model are primarily achieved through the regulation of immune and inflammatory responses, modulation of the extracellular matrix and signal transduction pathways, promotion of membrane repair, and enhancement of cellular antioxidant capacity. The PPI network analysis indicated that AJS-PDRN pretreatment may mitigate oxidative damage by promoting the expression of multiple selenoproteins in cells. In summary, H_2_O_2_ can cause cytotoxicity in RAW264.7 cells, resulting in oxidative damage, whereas AJS-PDRN can inhibit this damage through multiple pathways and is not cytotoxic. This study demonstrated the excellent antioxidant activity of AJS-PDRN and revealed its protective effect on oxidatively damaged RAW264.7 cells. This suggests that AJS-PDRN has significant potential in preventing and treating diseases caused by oxidative stress, particularly neurodegenerative diseases, cardiovascular diseases, and skin aging.

## Figures and Tables

**Table 1:** List of the 20 selected differentially expressed proteins in RAW264.7 cells.

Accession	Protein Description	Gene Symbol	H_2_O_2_/Control ^a^	PDRN/H_2_O_2_^b^
P01942	Hemoglobin subunit alpha	Hba	1.718	0.607
Q64669	NAD(P)H dehydrogenase [quinone] 1	Nqo1	1.641	0.515
Q9D0U6	Repressor of RNA polymerase III transcription MAF1 homolog	Maf1	2.329	0.035
P51667	Myosin regulatory light chain 2, ventricular/cardiac muscle isoform	Myl2	5.929	0.453
Q9CXT6	Bifunctional peptidase and arginyl-hydroxylase JMJD5	Kdm8	1.551	0.485
Q66JY6	Rho guanine nucleotide exchange factor 39	Arhgef39	1.546	0.578
Q64445	Cytochrome c oxidase subunit 8A, mitochondrial	Cox8a	0.069	1.979
P08905	Lysozyme C-2	Lyz2	0.647	0.581
Q05117	Tartrate-resistant acid phosphatase type 5	Acp5	0.404	0.463
O88199	Carbohydrate sulfotransferase 3	Chst3	45.981	67.64
Q9D722	Oxidative stress-responsive serine-rich protein 1	Oser1	10.169	16.966
P54987	Cis-aconitate decarboxylase	Acod1	0.392	0.571
O70167	Phosphatidylinositol 3-kinase C2 domain-containing subunit gamma	Pik3c2g	0.168	0.069
P05555	Integrin alpha-M	Itgam	0.638	0.642
Q9CPU9	Probable low-affinity copper uptake protein 2	Slc31a2	0.6	0.661
P11835	Integrin beta-2	Itgb2	0.663	0.661
Q61704	Inter-alpha-trypsin inhibitor heavy chain H3	Itih3	1.933	2.489
P54830	Tyrosine protein phosphatase non-receptor type 5	Ptpn5	0.408	0.464
O88531	Palmitoyl protein thioesterase 1	Ppt1	0.653	0.628
P31809	Carcinoembryonic antigen-related cell adhesion molecule 1	Ceacam1	0.543	0.538

^a^ Fold changes in the proteins in the H_2_O_2_ group vs. the control group. ^b^ Fold changes in the proteins in the AJS-PDRN group vs. the H_2_O_2_ group.

## Data Availability

The data are available from the corresponding author upon reasonable request.
